# Roles of the *PTP61F* Gene in Regulating Energy Metabolism of *Tribolium castaneum* (Coleoptera: Tenebrionidae)

**DOI:** 10.3389/fphys.2020.01071

**Published:** 2020-08-20

**Authors:** Kang-Kang Xu, Bi-Ying Pan, Yuan-Yuan Wang, Qian-Qian Ren, Can Li

**Affiliations:** ^1^Guizhou Provincial Key Laboratory for Rare Animal and Economic Insect of the Mountainous Region, Guizhou Provincial Engineering Research Center for Biological Resources Protection and Efficient Utilization of the Mountainous Region, College of Biology and Environmental Engineering, Guiyang University, Guiyang, China; ^2^College of Life and Environmental Sciences, Hangzhou Normal University, Hangzhou, China

**Keywords:** *Tribolium castaneum*, protein tyrosine phosphatase, RNA interference, trehalose metabolism, energy metabolism

## Abstract

Protein tyrosine phosphatase 1B (PTP1B) is a negative regulator in the insulin signaling pathway. It belongs to a class of non-receptor phosphatases of protein tyrosine phosphatase and can catalyze the dephosphorylation of tyrosine to regulate cell differentiation, growth, and metabolism. However, few studies have focused on the role of PTP1B in regulating energy metabolism of insects. In this study, we investigated the expression profiles and the functions of a *PTP1B* gene (designated *TcPTP61F*) in the red flour beetle *Tribolium castaneum*. Quantitative real-time PCR analyzed showed that *TcPTP61F* was highly expressed in the pupal and adult stages. In adult tissues, *TcPTP61F* was prominently expressed in the tarsus and head. RNA interference-mediated silencing of *TcPTP61F* reduced the expression of eight genes in trehalose metabolic and glycolytic pathways. *TcPTP61F* depletion also caused a significant change in the distribution of trehalose, glucose, and glycogen. Additionally, knockdown of *TcPTP61F* inhibited the pyruvate kinase (PK) activity and significantly decreased the adenosine triphosphate (ATP) level. The results suggest that *TcPTP61F* is indispensible for trehalose and energy metabolism of *T. castaneum*.

## Introduction

Insects need a continuous supply of energy to maintain their metabolism and activity. Energy metabolism in the insect body is constant. Insects directly use trehalose as their main energy source and it serves as the main sugar component of insect hemolymph (Yasugi et al., 2017). Trehalose is a highly stable, non-reducing disaccharide formed by two glucose molecules. It is found in a variety of organisms including bacteria, yeast, fungi, nematodes, insects, and some other invertebrates, but not in mammals (Elbein et al., 2003). In addition to playing a crucial role as an immediate source of energy, trehalose also plays important functions in insect response to stresses such as high or low temperatures, poor nutrition or starvation, oxidation, high osmotic pressure, toxic substances, and UV-B irradiation (Tamang et al., 2017; Chen J. X. et al., 2018). Under the catalysis of trehalose phosphate synthase (TPS), uridine diphosphate (UDP) glucose (UDP-glucose) and glucose-6-phosphate (G-6-P) synthesize trehalose-6-phosphate, which is then dephosphorylated under the action of 6-trehalose phosphate esterase (TPP) to produce trehalose. This is the most important path of trehalose synthesis in insects (Shukla et al., 2015; Tang et al., 2017; Chen J. X. et al., 2018).

When energy is needed, trehalose is hydrolyzed into glucose under the catalysis of trehalase (TRE), the only enzyme known to irreversibly degrade trehalose (Barraza and Sánchez, 2013). Glucose then enters the glycolysis-tricarboxylic acid (glycolysis-TCA) cycle. In the glycolysis pathway, glucose serves as the initial substrate and is converted into pyruvate by a series of enzymes, including hexokinase (HK), glucose-6-phosphate isomerase (G6PI), phosphofructokinase (PFK), and pyruvate kinase (PK) (Hu et al., 2016). Pyruvate can be further converted into acetyl-coenzyme A (acetyl-CoA), which can combine with oxaloacetic acid to enter TCA cycle and finally be oxidized to CO_2_, H_2_O, and adenosine triphosphate (ATP) (Hu et al., 2016). Insects could adapt to various physiological activities by regulating the rate of the glycolysis-TCA cycle. For example, some insects reduce the expression of glycolysis or TCA metabolic enzyme genes in the early pupal stage which saves energy for the development of new organs. These species include *Drosophila melanogaster*, *Bombyx mori*, and *Spodoptera litura* (White et al., 1999; Tian et al., 2010; Hu et al., 2016).

Insulin is important in regulating glucose homeostasis, lipid metabolism, and energy balance (White, 1998; Czech and Corvera, 1999). It can increase the transport of glucose and the synthesis of glycogen, diminish gluconeogenesis, inhibit glycogenolysis, and regulate the expression of many genes (Lochhead et al., 2001; Vital et al., 2010). Components of the insulin signaling pathway are extremely conserved in organisms as distantly related as humans, *D. melanogaster*, and *Caenorhabditis elegans* (Leevers, 2001). In *Aedes aegypti*, upstream components of the insulin signaling pathway, such as phosphatidylinositol 3-kinase (PI3K) (Pri-Tal et al., 2008) and protein kinase B (AKT) (Riehle and Brown, 2003), have been associated with glucose metabolism. The insulin signaling pathway not only controls the metabolic balance of blood glucose but also directly regulates juvenile hormones and ecdysone, which control insect development, metamorphosis, and reproduction (Satake et al., 1997; Vital et al., 2010; Kim and Hong, 2015).

Protein tyrosine phosphatases (PTPs, EC 3.1.3.48) are a large family of enzymes that regulate insulin signal transduction and are also involved in cell signal transduction and cell cycles (Klaman et al., 2000; Tonks, 2006). The PTP family is composed of four different subfamilies, classes I, II, III and IV (Sacco et al., 2012). Class I Cys-PTPs are the largest group of PTPs and could be divided into “classical” and dual specificity phosphatases, in which classical phosphatases are strictly devoted to the dephosphorylation of phosphotyrosine residues (Alonso et al., 2004). The family genes additionally participate in other physiological activities and metabolic processes such as cell differentiation, transformation, growth, reproduction, and immunity (Abaskharoun et al., 2010; Bao et al., 2011; Alho et al., 2013; Agouni et al., 2014). Protein tyrosine phosphatase 1B (PTP1B) is involved in the regulation of insulin action and other signal transduction pathways. PTP1B is a major regulator of energy balance, insulin sensitivity, and fat storage in insect body. In *Locusta migratoria tibetensis*, PTPN1-encoded insulin receptor inhibitor tyrosine protein phosphatase non-receptor type PTP1B is a negative regulator of the insulin pathway, and it plays an important role in response to hypoxic stress (Ding et al., 2018). The homologous gene of *PTP1B* in *D. melanogaster* encoded by PTP61F specifically targets to Dock and enhances the signal selectivity of insulin receptor (Wu et al., 2011).

The red flour beetle, *Tribolium castaneum* (Herbst) (Coleoptera: Tenebrionidae), is a worldwide pest of stored grains (Mehmood et al., 2018; Chen Q. W. et al., 2018). It has glands that secrete a liquid which causes a moldy smell in flour and this secretion also contains the carcinogen benzoquinone (Boateng and Obengofori, 2008). Control of *T. castaneum* in stored products and grain is primarily by fumigants and sprays, but insecticide resistance is now a major problem (Pimentel et al., 2010; Perkin and Oppert, 2019). Most importantly, *T. castaneum* is a model insect often used for research on gene function (Lorenzen et al., 2007). To date, few studies have evaluated the role of PTP1B in regulating energy metabolism in insects. In this present study, we identified and obtained a *PTP1B* gene (*TcPTP61F*) from *T. castaneum* and analyzed its expression patterns in different developmental stages and tissues. We used RNA interference *in vivo* to efficiently disrupt the *TcPTP61F* gene function in order to clarify its role in regulating the energy metabolism of *T. castaneum*.

## Materials and Methods

### Insect and Sample Preparation

The laboratory stock colony of *T. castaneum* were raised on whole wheat flour containing 5% yeast in an incubator at 28 ± 1°C and 65 ± 5% relative humidity under a constant 24 h dark (0L:24D).

In the tissue-specific experiment, the adults (male to female ratio 1:1) were used for tissue dissection. The antennae, head, wing, tarsus, epidermis, midgut, and fat body of *T. castaneum* were dissected under a stereomicroscope (Olympus SZX12, Tokyo, Japan). Pools of 200 individuals were used to prepare each body part. Samples at different developmental stages, including early larvae (EL, 2nd instar), middle larvae (ML, 5th instar), and late larvae (LL, 8th instar); early pupae (EP, 1st day), middle pupae (MP, 3rd day), and late pupae (LP, 5th day); and early adults (EA, 1st day), middle adults (MA, 3rd day), and late adults (LA, 5th day) were collected separately. *T. castaneum* used for the microinjection of double-stranded RNA (dsRNA) were the 1st day of the 8th instar larvae, and the insects at 48 and 72 h after injection were collected for determination of gene expression, carbohydrate content, and ATP content. Ten individuals were used for gene expression, and 20 for carbohydrate, and ATP contents, respectively. All above the samples were immediately frozen in liquid nitrogen and stored at -80°C. All of the experiments were repeated three times.

### RNA Isolation and cDNA Synthesis

Total RNA was isolated from each sample by using the MiniBEST Universal Extraction Kit (TaKaRa, Dalian, China), following the manufacturer’s instructions. Total RNA integrity was evaluated using 1% agarose gel electrophoresis, and the RNA concentration and purity were determined by a NanoDrop 2000C Spectrophotometer (Thermo Fisher Scientific, Waltham, MA, United States). First-strand complementary DNA (cDNA) synthesis was performed using the PrimeScript^®^ RT Reagent Kit (TaKaRa, Dalian, China) following manufacturer’s instructions.

### Synthesis and Injection of dsRNA

RNAi was used to study the potential function of *TcPTP61F* in *T. castaneum*. The dsRNA primers (Table 1) for *TcPTP61F* and green fluorescent protein (*GFP*, as control) were designed using E-RNAi^1^. The PCR products were subject to T cloning, followed by a subsequent amplification with primers containing the T7 promoter sequence. Cross-PCR reactions were performed using a T7 RiboMAX^TM^ Express RNAi System kit (Promega, Madison, WI, United States) to synthesize dsRNA. The dsRNA was diluted with nuclease-free water to a final concentration of 2 μg/μL. Using a Nanoliter 2010 injector (World Precision Instruments, Sarasota, FL, United States), 200 ng of ds*TcPTP61F* or ds*GFP* was slowly injected into the hemocoel between the third and fourth abdominal segments of each 1st day of the 8th instar larvae. Three biological replicates (each with at least 50 larvae) were treated by ds*TcPTP61F* and ds*GFP* injection. The insects treated with dsRNA were reared under the same conditions as mentioned above.

**TABLE 1:** Primers used to synthesize dsRNA and analyze transcript levels.

Application of primers	Gene name	Forward primer (5′–3′)	Reverse primer (5′-3′)	Length (bp)
dsRNA synthesis	*TcPTP61F*	T7-GTCATCGGGCAATAACATC	T7-ATATCTCGGGACTCTTTCGT	584
	*GFP*	T7-AAGGGCGAGGAGCTGTTCACCG	T7-CAGCAGGACCATGTGATCGCGC	256
qPCR analysis	*TcPTP61F*	CCAAATATCCCCAAGAGC	GACTATCGGAACGCAAATC	151
	*TcTre1-1*	AACGACTCGCAATGGCTGG	CGGAGGCGTAGTGGAATAGAG	127
	*TcTre1-2*	GTGCCCAATGGGTTTATCG	CAACCACAACACTTCCTTCG	261
	*TcTre1-3*	CCTCTCATTCGTCACAAGCG	AAGCGTTTGATTTCTTTGCG	205
	*TcTre1-4*	ACGGTGCCCGCATCTACTA	GTGTAGGTGGTCCCGTTCTTG	187
	*TcTre2*	CTCAGCCTGGCCCTTAGTTG	GGAGTCCTCGTAGATGCGTT	120
	*TcTPS*	CGATTCGTACTACAACGGCTGC	GTGGTGTAGCATTGCCAGTGC	105
	*TcHK1*	CGCACCGAATGCCAGAATC	GACCCACCCGACATCGATT	141
	*TcHK2*	CGAATCGGCCTAATAGTTGGC	GACGGAGCCCTCGATTTCAT	155
	*TcG6PI*	GTGATGCCGGAGGTGAAT	CACGTCGGTGATGGGCTT	112
	*TcPK*	CAATTTTACCGCATCTCAAC	GTCTCCATCATTTTCTCCAAC	240
	*TcRPL13a*	ACCATATGACCGCAGGAAAC	GGTGAATGGAGCCACTTGTT	250

**T7:GGATCCTAATACGACTCACTATAGG*.*

### Quantitative Real-Time Polymerase Chain Reaction (qPCR)

The qPCR was conducted to confirm the relative expression levels of *PTP61F* in various developmental phases and tissues. At 48 and 72 h after dsRNA injection, the insects from each treatment were collected for qPCR to assess the efficiency of the RNAi. After *PTP61F* was knocked down, transcript levels of six trehalose metabolic pathway genes, including five trehalases (*TcTre1-1*, *TcTre1-2*, *TcTre1-3*, *TcTre1-4*, and *TcTre2*) and trehalose-6-phosphate synthase gene (*TcTPS*); and glycolytic pathway genes, such as hexokinases (*TcHK1* and *TcHK2*) and glucose-6-phosphate isomerase (*TcG6PI*); and pyruvate kinase gene (*TcPK*) were detected by qPCR after injection. The qPCR was carried out on a CFX-96 real-time detection system (Bio-Rad, Hercules, CA, United States) in a 20 μL reaction containing 1 μL (100 ng/μL) cDNA, 1 μL (10 μM) each primer (Table 1), 7 μL nuclease-free water, and 10 μL of GoTaq^®^ qPCR MasterMix (Promega). The reaction was performed under the following conditions: pre-incubation at 95°C for 2 min, followed by 40 cycles of 95°C for 30 s and annealing at 60°C for 30 s, with a melting curve at 65–95°C. Amplification of *Ribosomal Protein L13a* (*RPL13a*) was used as an internal control. All of the experiments were performed in triplicate, with two technical replicates each. The 2^–△△CT^ method was used for the analysis of relative gene expression (Livak and Schmittgen, 2001).

### Analysis of Trehalose, Glucose, and Glycogen Contents

Twenty individuals, collected after injection, were used to measured trehalose, glucose and glycogen contents. The anthrone-sulfuric acid method was used to measure the trehalose content (Leyva et al., 2008). The assay of glucose and glycogen contents was performed according to the previously described methods (Zhang et al., 2017). Three independent biological replicates were used for assays. Briefly, the samples were homogenized in 200 μL phosphate buffer saline (PBS; pH 7.0), then 800 μL PBS was added up to 1 mL. Subsequently, the homogenate was centrifuged at 1,000 g for 20 min at 4°C. The supernatant (300 μL) was taken to detect concentrations of protein, trehalose, and glycogen as described below. Then 350 μL of the supernatant was removed and ultracentrifuged at 20,800 g for 60 min at 4°C. The supernatant (300 μL) obtained from ultracentrifugation was used to determine the concentration of protein and glucose. The sediment was suspended in PBS (300 μL) then used for the determination of protein and glucose contents. The glucose content was determined using a glucose (GO) Assay Kit (Sigma-Aldrich, St. Louis, MO, United States) according to manufacturer instructions. As for the determination of glycogen content, the methods were similar to the methods of glucose content measurement unless the samples required 4 h decomposition reaction by amyloglucosidase (catalog no. 10115, Sigma-Aldrich). The protein content was determined using the BCA Protein Assay Kit (Beyotime, China).

### Determination of PK Activity and Measurement of ATP Content

The beetles were mixed into physiological saline (g:mL = 1:9) for grinding and crushing to obtain a 10% homogenate. The 10% homogenate was prepared with physiological saline, and the experiment was conducted according to the instruction on the Pyruvate Kinase Assay Kit (No. A076-1-1, Nanjing Jiancheng Bioengineering Institute) to determinate the PK activity. Measurement of ATP content was determined according to the instruction of ATP Assay Kit (No. A095-1-1, Nanjing Jiancheng Bioengineering Institute).

### Statistical Analyses

All of the data were presented as mean ± standard error (SE) and were analyzed using SPSS version 20 software (SPSS Inc., Chicago, IL, United States). A one-way analysis of variance (ANOVA) followed by a least significant difference (LSD) test was used for comparing the differences among more than two samples. Differences between two groups were compared using Student’s *t*-test (*P < 0.05, **P < 0.01).

## Results

### The Developmental and Tissue Expression Profiles of *TcPTP61F*

**FIGURE 1:**
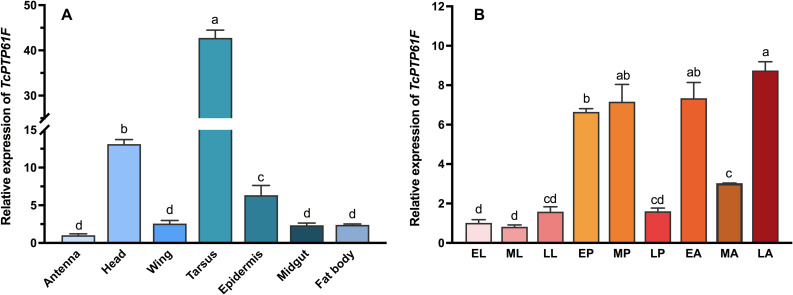
Relative expression levels of *TcPTP61F* in different tissues **(A)** and different developmental stages **(B)** of *Tribolium castaneum*. EL, ML, LL, EP, MP, LP, EA, MA, and LA represent early instar larvae, middle instar larvae, late instar larvae, early pupae, middle pupae, late pupae, early adults, middle adults, and late adults, respectively. Different letters above bars indicate significant differences based on one-way ANOVA followed by a least significance difference test (*P* < 0.05).

We measured the expression level of *TcPTP61F* in the antenna, head, wing, tarsus, epidermis, midgut, and fat body from adult *T. castaneum* to study tissue-specific expression profiles of *TcPTP61F* (Figure 1A). Among the seven tissues, the highest expression level of *TcPTP61F* was detected in the tarsus, followed by the head and epidermis; expression was relatively lower in the other tissues. As for developmental expression profiles illustrated in Figure 1B, the mRNA of *TcPTP61F* was highly expressed in the pupal and adult stages was stable at a low expression level in larvae. The results showed that the expression of *TcPTP61F* varied across developmental stages and among many tissues.

### Evaluation of the Efficiency of RNAi Knockdown by ds*TcPTP61F*

**FIGURE 2:**
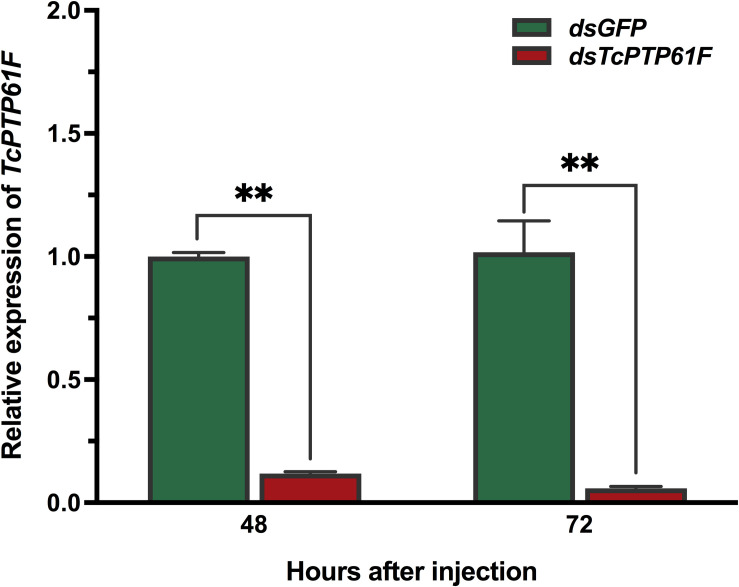
Relative expression levels of *TcPTP61F* at 48 and 72 h after *TcPTP61F* or *GFP* dsRNA injection. Significant differences between the RNAi group and the control group were determined using Student’s *t*-test (**p < 0.01).

To determine the effect of RNAi, the relative expression level of *TcPTP61F* were detected by qPCR. The results showed that the expression level of *TcPTP61F* was significantly decreased at 48 and 72 h after ds*TcPTP61F* injection (*P* < 0.01) (Figure 2), and the interference efficiency were 88.14 and 94.29%, respectively, indicating the successful inhibition of the target genes.

### Effects on the Relative Expression Levels of Genes in the Trehalose Metabolic Pathway Following *TcPTP61F* Knockdown

**FIGURE 3:**
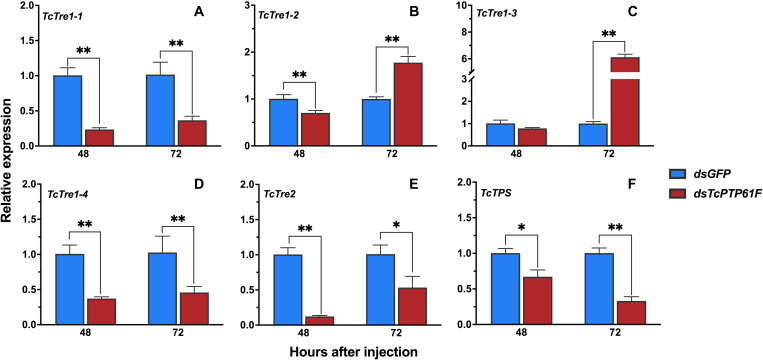
Effects of *TcPTP61F* knockdown on the expressions of six trehalose metabolic pathway genes. The relative expression levels of five trehalases (*TcTre*, **A–E**) and one trehalose-6-phosphate synthases (*TcTPS*, **F**) at 48 and 72 h after *TcPTP61F* or *GFP* dsRNA injection. The expression values were calculated by comparison to the ds*GFP* group, which was normalized at 1. Significant differences were identified by Student’s *t*-test (*P < 0.05, **P < 0.01).

The results showed that the mRNA levels of *TcTre1-1*, *TcTre1-4*, *TcTre2*, and *TcTPS* decreased significantly at 48 and 72 h after *TcPTP61F* was inhibited (*P* < 0.05) (Figures 3A,D–F). The expression levels of *TcTre1-2* and *TcTre1-3* decreased at 48 h but increased significantly at 72 h after injection of ds*TcPTP61F* (*P* < 0.01) (Figures 3B,C).

### Effects on the Content of Trehalose, Glucose, and Glycogen Following ds*TcPTP61F* Injection

**FIGURE 4:**
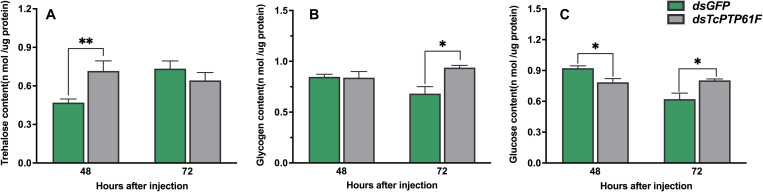
Effects of *TcPTP61F* knockdown on content of trehalase **(A)**, glycogen **(B)**, and glucose **(C)**. Significant differences were identified by Student’s *t*-test (*P < 0.05, **P < 0.01).

The content of trehalose increased 52.62% at 48 h after injection with ds*TcPTP61F* (*P* < 0.01) and then was restored to a normal level at 72 h compared to the ds*GFP* group (Figure 4A). In contrast, the glycogen and glucose contents significantly increased at 72 h after *TcPTP61F* was knocked down (*P* < 0.05) (Figures 4B,C). The glycogen content was stable at 48 h after ds*TcPTP61F* injection (Figure 4B), whereas the glucose content decreased significantly (*P* < 0.05) (Figure 4C).

### Effects of *TcPTP61F* Knockdown on the Relative Expression of Critical Genes in the Glycolytic Pathway

**FIGURE 5:**
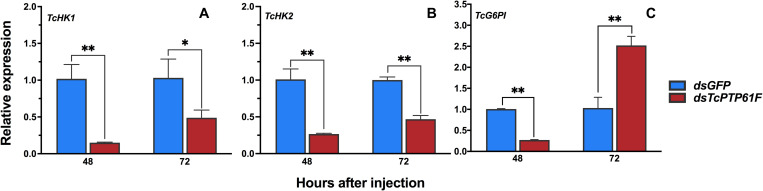
Effects of *TcPTP61F* knockdown on the expressions of three glycolytic pathway pathway genes. The relative expression levels of *TcHK1* **(A)**, *TcHK2* **(B)**, and *TcG6PI* **(C)** at 48 and 72 h after *TcPTP61F* or *GFP* dsRNA injection. Significant differences were identified by Student’s *t*-test (*P < 0.05, **P < 0.01).

After *TcPTP61F* was knocked down, the relative expressions of *HK1* and *HK2* were significantly downregulated at 48 and 72 h (*P* < 0.01) (Figures 5A,B). In addition, the relative expression of *G6PI* was significantly downregulated at 48 h, but upregulated sharply at 72 h (*P* < 0.01) (Figure 5C).

### Effects on the Enzyme Activity of PK and ATP Content After *TcPTP61F* Knockdown

**FIGURE 6:**
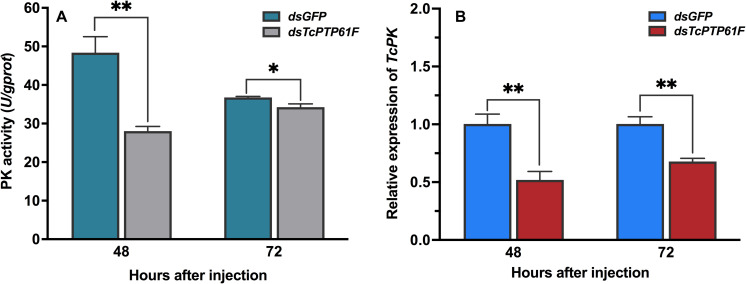
Effect of *TcPTP61F* knockdown on pyruvate kinase activity **(A)** and expression of *TcPK* gene **(B)**. Significant differences were identified by Student’s *t*-test (^∗^*P* < 0.05, ^∗∗^*P* < 0.01).

**FIGURE 7:**
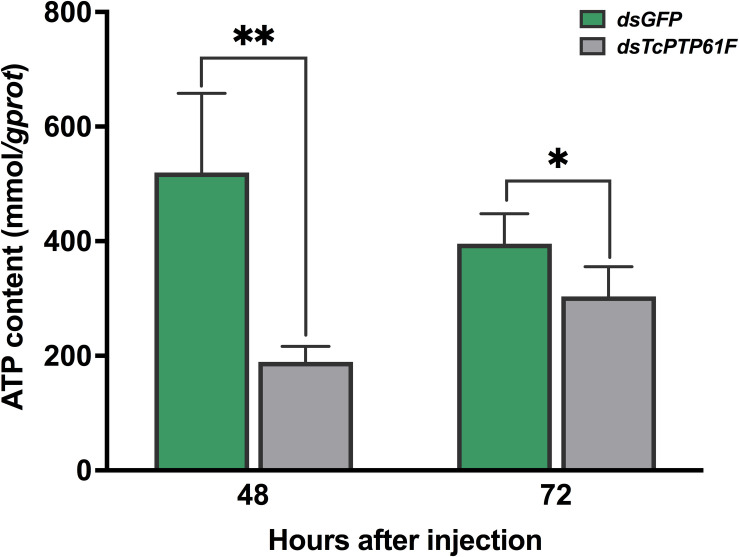
Effect of *TcPTP61F* knockdown on content of adenosine triphosphate (ATP). Significant differences were identified by Student’s *t*-test (*P < 0.05, **P < 0.01).

When *TcPTP61F* was inhibited, the enzyme activity of PK significantly decreased at 48 and 72 h, especially it was decreased at 48 h by 42.03% compared to the ds*GFP* group (*P* < 0.05) (Figure 6A). Consistently, the mRNA level of *TcPK* was significantly down-regulated at 48 and 72 h after *TcPTP61F* suppression (*P* < 0.01) (Figure 6B). The ATP content decreased 63.61 and 23.15% at 48 and 72 h after ds*TcPTP61F* injection, respectively (Figure 7).

## Discussion

Protein phosphorylation and dephosphorylation is one of the most important biochemical reactions in the body and plays a role in regulating cell growth, proliferation, differentiation, and immunity (Mustelin et al., 2005; Tonks, 2006). Protein tyrosine phosphatase is a type of superfamily phosphatase that changes its phosphate by specifically catalyzing the removal of phosphate groups on phosphorylated modified tyrosine residues to control cell function (Tonks, 2006). Although the amino acid sequences and substrates of PTPs differ, most PTPs have a conserved structural motif (Motif) (H/V) C (X) R (S/T) (Supplementary Figure S1), and this structural phantom plays a key role in the catalytic activity of PTP (Wang et al., 2014). The amino acid sequences of PTP1B in several insect species contain a catalytic domain of protein tyrosine phosphatase (PTPc) (Supplementary Figure S2).

PTP1B is a ubiquitously expressed intracellular protein tyrosine phosphatase (Forsell et al., 2000). It is common in the testis, kidney, spleen, muscle, heart, liver, and brain of mice (Miyasaka and Li, 1992). However, there are few studies of PTP1B in insects. In *A. aegypti*, it has been reported to occur in all tissues, and it is highly expressed in the ovaries (Moretti et al., 2014). In this study, we found that *TcPTP61F* is expressed in a variety of *T. castaneum* tissues. This study did not evaluate the *T. castaneum* ovary but it showed that *TcPTP61F* was highly expressed in the tarsus and the head. Muscle has been revealed as one of the insulin’s key target tissues (Saltiel and Kahn, 2001; Biddinger and Kahn, 2006), and it is known that tarsus contains a great number of muscle, which may be the main reason of the high expression of *TcPTP61F* in tarsus. Like the mosquito, the head of *T. castaneum* is also composed of many different structures and tissues, and responsible for several physiological processes (Predel et al., 2010). Meanwhile, phosphorylation of tyrosine residues induces phosphotyrosine (pTyr) signaling pathways are important signal transduction systems for many cellular functions such as protein synthesis and cell proliferation. Further studies are required to clarify the roles of *TcPTP61F* in the tarsus and head of *T. castaneum*. In the developmental pattern of *TcPTP61F*, we found relatively lower expression in the larval stage, but higher expression in the pupal and adult stages, which demonstrates differences in *TcPTP61F* at different developmental stages. A transient increase occurred in the total tyrosine phosphorylation of the *A. aegypti* head during the first days after adult emergence (Jablonka et al., 2011), and our results are consistent with the those in *A. aegypti*.

PTP1B is a member of the PTPs family and plays a key regulatory role in insulin and leptin signaling. By catalyzing the dephosphorylation of phosphotyrosine (pTyr), it maintains the level of phosphorylation of protein tyrosine together with protein tyrosine kinase (PTKs) (Tiganis and Bennett, 2007). PTP1B regulates the insulin signaling pathway with tissue specificity. The lack of T-cell protein tyrosine phosphatase (TCPTP) in the muscles of mice does not alter insulin signaling and glucose homeostasis (Loh et al., 2012). RNAi is an effective means to inhibit gene expression through dsRNA injection and it is widely used to study gene function (Prentice et al., 2017). To explore the potential role of *TcPTP61F* in *T. castaneum*, we knocked down *TcPTP61F* using RNAi technology. The expression of *TcPTP61F* decreased significantly at 48 and 72 h following ds*TcPTP61F* injection. Trehalose can provide energy to promote development, metamorphosis, stress recovery, chitin synthesis, and flight (Wegener et al., 2003, 2010; Tatun et al., 2014; Shukla et al., 2015; Tang et al., 2017; Zhang et al., 2017). In addition, trehalase is critical to the role of trehalose in insect physiology because it is required for regulation of metabolism and glucose generation (Shukla et al., 2015). Therefore, we tested the gene expression of the trehalose metabolism pathway of *T. castaneum* after RNAi and found that almost all *Tre* and *TPS* genes were significantly downregulated. However, *TcTre1-2* and *TcTre1-3* increased at 72 h after injection. These results suggest that the inhibition of *TcPTP61F* effected the regulation of the trehalose metabolism pathway. To further analyze its effect on trehalose metabolism, we tested the trehalose content and found that the synthesis of trehalose increased significantly at 48 h and returned to normal levels at 72 h (Figure 4A). This finding may be the result of the conversion of these three kinds of sugars. From the results of glucose and glycogen, we observed that trehalose accumulated at 48 h and was converted into glucose and glycogen after 72 h, resulting in a significant increase in glycogen and glucose content at 72 h (Figures 4B,C). The increased expression of *TcTre1-2* and *TcTre1-3* might be main cause of the destruction of glucose hemostasis. These results all indicate that the silencing of *TcPTP61F* can have an effect on trehalose metabolism.

Glycolysis is an important method of energy metabolism and it involves the process of glucose decomposed into pyruvate, under an anaerobic environment, by a series of enzymes (Sunna et al., 1997). Among these enzymes, hexokinase (HK) is the first rate-limiting enzyme in the glycolytic pathway, which plays a key role in glucose homeostasis and energy metabolism through glucose (Glc) phosphorylation and Glc signaling (Ge et al., 2019). Therefore, we tested the expression of *HK* to investigate the effect of ds*TcPTP61F* injection on the glucose homeostasis and energy metabolism of *T. castaneum*. The results showed that the expression of two *TcHK* genes in *T. castaneum* was significantly downregulated (Figures 5A,B). However, the glucose content decreases significantly at 48 h, it may be due to less glucose being synthesized. Furthermore, the glucose content increased significantly after RNAi at 72 h, which may be caused by the decomposition of a large amount of trehalose.

The PK enzyme catalyzes the conversion of phosphoenolpyruvate and ADP to pyruvate and ATP in glycolysis, and pyruvate can be further converted into acetyl-CoA that can combine with oxaloacetic acid to enter the TCA cycle (Israelsen and Vander Heiden, 2015). The TCA cycle, together with the subsequent electron transport chain, is one of the main metabolic pathways that provides energy to support cellular homeostasis under aerobic conditions (Gaster et al., 2012). Our results showed that PK enzyme activity was significantly reduced after *TcPTP61F* knockdown. Adenosine triphosphate has a fundamental intracellular role and is the direct energy source of most activities in living cells (Bodin and Burnstock, 2001). In this study, after *TcPTP61F* knockdown, the ATP content was significantly decreased both at 48 and 72 h.

**FIGURE 8:**
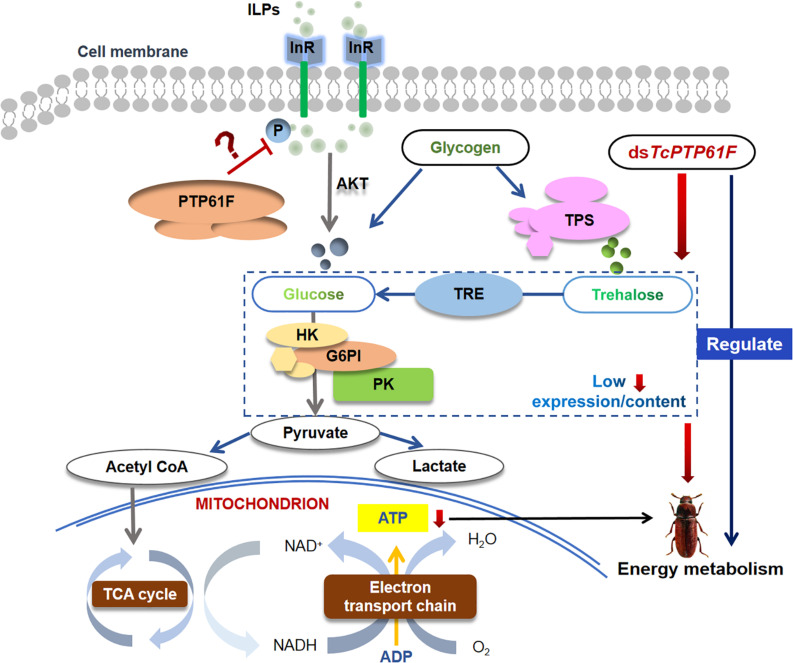
Schematic description of the hypothesis for the roles of *TcPTP61F* in regulating energy metabolism of *T. castaneum*. PTP61F acts as insulin receptor inhibitor causes changes in the distribution of trehalose, glucose, and glycogen as well as a decreased glycolysis level. Downregulation of *TcPTP61F* caused a decreased ATP content. The inhibition of *TcPTP61F* resulted in disorders of energy metabolism in *T. castaneum.*

Based on our results and previous studies, we propose a hypothesis for the roles of *TcPTP61F* in regulating energy metabolism of *T. castaneum* (Figure 8). PTP61F acts as insulin receptor inhibitor that causes changes in the distribution of trehalose, glucose, and glycogen as well as a decreased glycolysis level. Downregulation of *TcPTP61F* caused a decreased ATP content. The inhibition of *TcPTP61F* resulted in disorders of energy metabolism in *T. castaneum*.

## Data Availability Statement

The raw data supporting the conclusions of this article will be made available by the authors, without undue reservation, to any qualified researcher.

## Author Contributions

K-KX, B-YP, and CL conceived and designed the experiments, and wrote the manuscript. B-YP, Y-YW, and Q-QR performed the experiments. CL revised the manuscript. All authors gave final approval for the publication. All authors contributed to the article and approved the submitted version.

## Conflict of Interest

The authors declare that the research was conducted in the absence of any commercial or financial relationships that could be construed as a potential conflict of interest.

## Footnotes

1 http://www.dkfz.de/signaling/e-rnai3/idseq.php

## Supplementary Material

FIGURE S1 Multiple alignments of the amino acid sequence of *TcPTP61F* with homologs from other insect species. Identical and similar amino acids are marked with the same color. Gaps have been introduced to permit alignment. The heights of the pink bars below the aligned sequences represent the degree of similarity of the amino acids. The red box indicate the conserved catalytic cysteine. TcPTP61F (*Tribolium castaneum*; GenBank accession number: XP_008190655.1), DmPTP61F (*Drosophila melanogaster*; NP_476688.1), AgPTP61F1 (*Anoplophora glabripennis*; XP_018577682.1), DvPTP61F1 (*Diabrotica virgifera*; XP_028144768.1), LmPTPN1 (*Locusta migratoria*; AYI50186.1), LdPTP61F1(*Leptinotarsa decemlineata*; XP_023013201.1), AtPTP61F1(*Aethina tumida*; XP_019879072.1), NvPTP61F1(*Nicrophorus vespilloides*; XP_017775944.1), and OtPTP61F1(*Onthophagus taurus*; XP_022904338.1).

FIGURE S2 Domain organization of PTP61Fs from *T. castaneum* and other insect speicies. The domain organization was generated with the SMART tool (http://smart.embl-heidelberg.de/) by using the protein sequences. The dark blue frame indicate the protein tyrosine coiled-coiloscioled-coliatase, catalytic domain (PTPc), and the light blue frame indicates the amino acid responsible for the recognition of Y-coiled-coiloscioled-coliatase. Structure of the coiled-coil and transmembrane region of PTP61Fs are represented as yellow ellipse and green polygon, respectively.
