# Supplementary figures and images for: Roles of the PTP61F Gene in Regulating Energy Metabolism of Tribolium castaneum (Coleoptera: Tenebrionidae)
Source: Front Physiol. 2020 Aug 20;11:1071. doi: 10.3389/fphys.2020.01071 (PMC7468486; doi:10.3389/fphys.2020.01071)

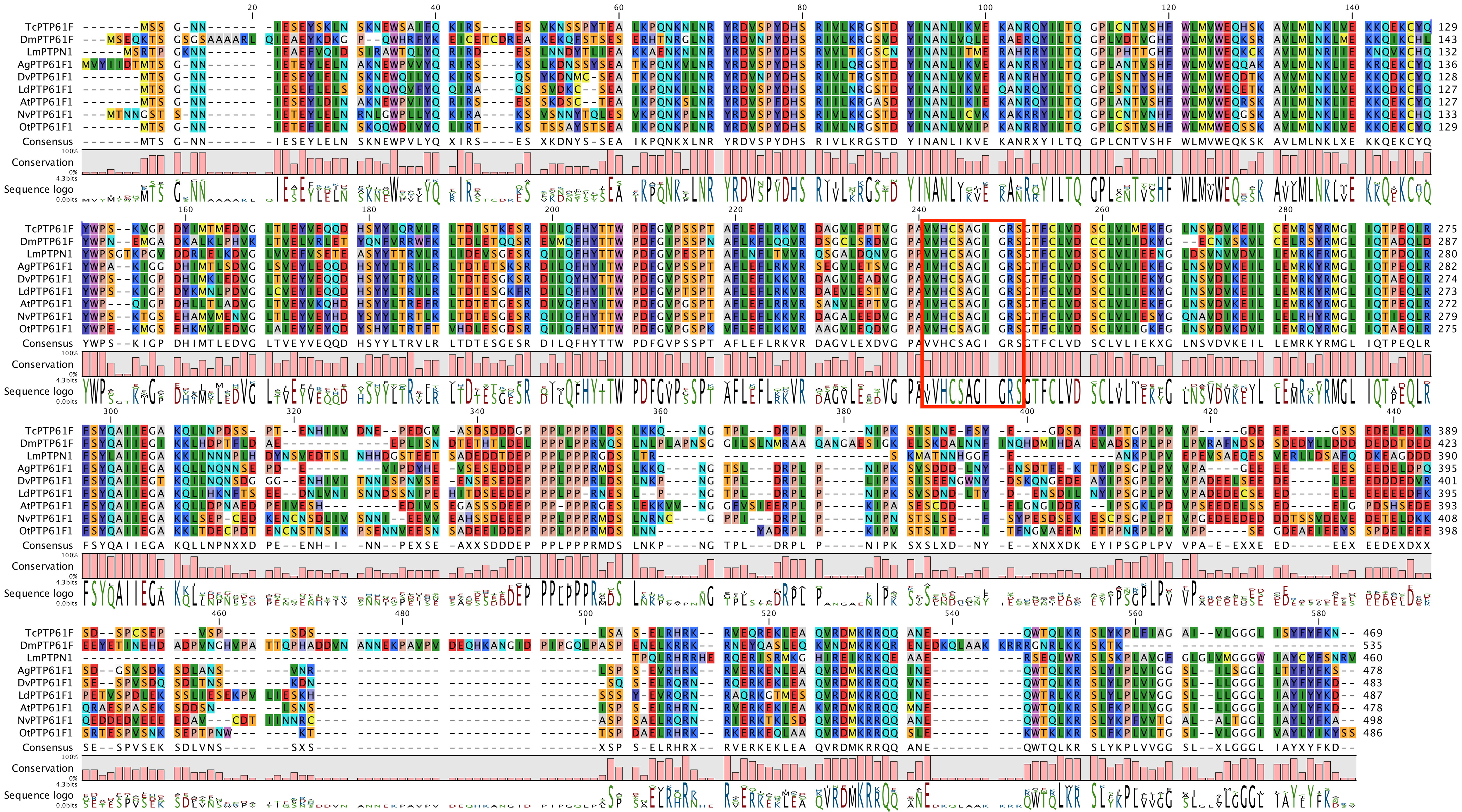

Supplement: FIGURE S1 — Multiple alignments of the amino acid sequence of TcPTP61F with homologs from other insect species. Identical and similar amino acids are marked with the same color. Gaps have been introduced to permit alignment. The heights of the pink bars below the aligned sequences represent the degree of similarity of the amino acids. The red box indicate the conserved catalytic cysteine. TcPTP61F (Tribolium castaneum; GenBank accession number: XP_008190655.1), DmPTP61F (Drosophila melanogaster; NP_476688.1), AgPTP61F1 (Anoplophora glabripennis; XP_018577682.1), DvPTP61F1 (Diabrotica virgifera; XP_028144768.1), LmPTPN1 (Locusta migratoria; AYI50186.1), LdPTP61F1(Leptinotarsa decemlineata; XP_023013201.1), AtPTP61F1(Aethina tumida; XP_019879072.1), NvPTP61F1(Nicrophorus vespilloides; XP_017775944.1), and OtPTP61F1(Onthophagus taurus; XP_022904338.1). [file Image_1.JPEG]

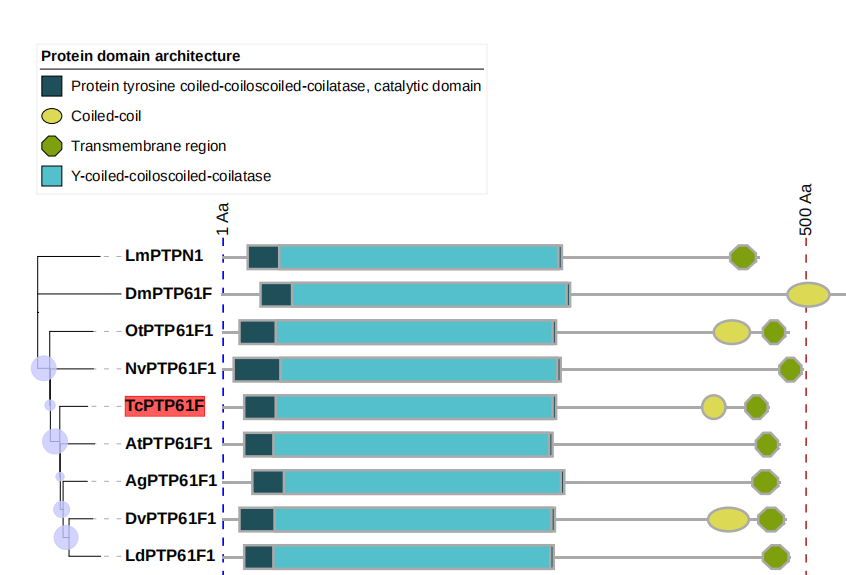

Supplement: FIGURE S2 — Domain organization of PTP61Fs from T. castaneum and other insect speicies. The domain organization was generated with the SMART tool (http://smart.embl-heidelberg.de/) by using the protein sequences. The dark blue frame indicate the protein tyrosine coiled-coiloscioled-coliatase, catalytic domain (PTPc), and the light blue frame indicates the amino acid responsible for the recognition of Y-coiled-coiloscioled-coliatase. Structure of the coiled-coil and transmembrane region of PTP61Fs are represented as yellow ellipse and green polygon, respectively. [file Image_2.TIF]
